# Supplementary material for: Archaic introgression and the distribution of shared variation under stabilizing selection
Source: PLoS Genet. 2025 Mar 31;21(3):e1011623. doi: 10.1371/journal.pgen.1011623 (PMC11964463; doi:10.1371/journal.pgen.1011623)
Supplement: S1 Text — (PDF) [file pone.1011623.s001.pdf]

Supporting Information for "Archaic introgression and the distribution of shared variation under stabilizing selection"

Aaron P. Ragsdale

Department of Integrative Biology, University of Wisconsin–Madison, WI, USA

March 3, 2025

A Stabilizing selection results in selection on trait-affecting alleles equivalent to symmetric underdominance

This result is well known and has been derived many times before. We include it here for completeness. We consider a polygenic trait under stabilizing selection, and we assume the mean phenotype of the population is close to the optimal value (0), which will be the case if the optimum has not shifted recently. Because many segregating loci are assumed to contribute to the phenotypic variance of the trait, the phenotypic distribution is well-approximated as normally distributed around the optimum: $f(G) = \frac{1}{\sqrt{2\pi V_G}} e^{-G^2/2V_G}$. We assume a Gaussian fitness function, so that the fitness of an individual with genotypic value G is given by $w(G) = e^{-G^2/2V_S}$.

For a derived allele (labeled 1) with frequency p, the expected change in frequency over one generation due to selection (in general) is

$$\mathbb{E}[\Delta p] = p(1-p)\frac{w_{1\cdot} - w_{0\cdot}}{\bar{w}},$$

where $\bar{w}$ is the mean fitness of the population, and marginal fitnesses for the derived and ancestral alleles are

$$w_{1\cdot} = pw_{11} + (1-p)w_{01},$$

and

$$w_{0\cdot} = pw_{01} + (1-p)w_{00}.$$

Here, w_{11}, w_{01}, and w_{00} are the relative fitnesses of individuals who are homozygous for the derived allele, heterozygous, or homozygous for the ancestral allele, respectively.

We partition an individual's genetic values into contributions from their genetic background ($\tilde{G}$) and the focal locus: $G = \tilde{G} + xa$, where $x \in 0, 1, 2$. We then integrate over genetic backgrounds to find the expected fitnesses of each genotype. As described in SI section 2.1 of [1], because each individual locus contributes a small amount to V_G, $V_{\tilde{G}} \approx V_G$ and the fitness function is well approximated as $f(\tilde{G}) = \frac{1}{\sqrt{2\pi V_G}} e^{-\tilde{G}^2/2V_G}$. Then, mean fitness

$$\bar{w} = \sqrt{\frac{V_S}{V_S + V_G}},$$

as shown in the main text, and

$$w_{11} \approx \int_{-\infty}^{\infty} f(\tilde{G}) w(\tilde{G} + 2a) d\tilde{G} = \bar{w} \exp\left(\frac{-4a^2(1-p)^2}{2(V_S + V_G)}\right),$$

$$w_{01} \approx \int_{-\infty}^{\infty} f(\tilde{G}) w(\tilde{G} + a) d\tilde{G} = \bar{w} \exp\left(\frac{-a^2(1-2p)^2}{2(V_S + V_G)}\right),$$

and

$$w_{00} \approx \int_{-\infty}^{\infty} f(\tilde{G}) w(\tilde{G}) d\tilde{G} = \bar{w} \exp\left(\frac{-4a^2p^2}{2(V_S + V_G)}\right).$$

Taking the first-order Taylor series expansion of the exponentials ($e^{-x} \approx 1-x$, for small x) and combining terms,

$$\frac{w_{1\cdot} - w_{0\cdot}}{\bar{w}} \approx \frac{-a^2(1-2p)}{2(V_S + V_G)},$$

demonstrating the result.

This same result can be found by directly considering the marginal fitness of a focal allele against the expected haploid background, as pointed out by [2]. Thus, while the selection coefficient takes an equivalent form to underdominance, we note that the mechanism differs: selection acts against the minor allele, rather than against the heterozygous genotype.

B Moment equations for over- and underdominance

For symmetric underdominance, the relative fitnesses of genotypes $aa : Aa : AA$ are $1 : 1-t : 1$. We consider any value of t (positive or negative, corresponding to either under- or overdominance); with stabilizing selection, $t = \frac{a^2}{2V_S}$, so that heterozygous individuals always have reduced fitness compared to either homozygote.

We extended `moments` [3] to compute the sample site-frequency spectrum $\Phi_{\mathbf{n}}$ for one or more (up to five) populations with sample sizes $\mathbf{n}$. This provides a good approximation for the distribution of trait-affecting allele frequencies across multiple populations if the trait optimum is shared across populations and each population's mean phenotype remains close to that optimum. This is expected to be the case if there are no optimum shifts in any lineage. Hardy-Weinberg equilibrium is assumed at all loci. Accounting for optimum shifts in one or more lineages would require a combination of direct and underdominant selection [e.g., 4], which we leave for future work.

We refer readers to [3] for a detailed introduction to the general moments-based framework for the dynamics of $\Phi_{\mathbf{n}}$. Here, we describe how underdominant selection changes Φ over a single generation. With symmetric underdominance, selection acts on heterozygotes. This can be formulated as some proportion of heterozygotes failing to reproduce in a given generation, with those selected lineages replaced by copies drawn from the full population.

In a single population, we consider n tracked lineages of which i of those carry the derived allele ($\Phi_n(i)$ is thus the count of loci with i observed derived alleles in a haploid sample of size of n). i can increase or decrease due to selection "events". Here, we assume t is small enough so that at most a single selection event occurs among the n lineages in any given generation. This is a reasonable approximation as long as t is not extremely large [3, 5] – for selection coefficients induced by stabilizing selection, this is typically a safe assumption.

Two selective events can change i to $i+1$ or $i-1$: (a) a tracked copy carrying a derived allele is heterozygous (paired with an ancestral allele-carrying copy), selected against, and then replaced by an ancestral allele drawn from the rest of the population, so that $i \to i-1$, or (b) a tracked copy carrying the ancestral allele is paired with a derived allele-carrying copy, selected against, and then replaced by a derived allele, so that $i \to i+1$. In both cases, we require drawing two additional lineages to find the Φ_n in the next generation, one for the diploid pair and one for the replacement allele (i.e., $\Phi_n^{t+1}(i)$ requires Φ_{n+2}^t). This results in an unclosed system of equations, and we use a quadratic jackknife approximation to approximate Φ_{n+2} from Φ_n, as described in [3].

The expected increase and decrease of $\Phi_n(i)$ due to selection is found by enumerating the sampling probabilities of each selective event. For case (a), $i \to i-1$ (i.e., $\Phi_n(i)$ is reduced) with probability

$$t\frac{i(n-i+2)(n-i+1)}{(n+2)(n+1)}\Phi_{n+2}(i),$$

and $i+1 \to i$ ($\Phi_n(i)$ increases) with probability

$$t\frac{(i+1)(n-i+1)(n-i)}{(n+2)(n+1)}\Phi_{n+2}(i+1).$$

For case (b), $i \to i+1$ with probability

$$t\frac{(n-i)(i+2)(i+1)}{(n+2)(n+1)}\Phi_{n+2}(i+2),$$

and $i-1 \to i$ with probability

$$t\frac{(n-i+1)(i+1)i}{(n+2)(n+1)}\Phi_{n+2}(i+1).$$

These can be combined (with negative rates for the reduction of $\Phi_n(i)$) to describe the change of $\Phi_n(i)$ for all $0 \leq i \leq n$. Figs O–Q show that this approach, implemented in `moments`, is accurate compared to discrete Wright-Fisher simulations.

C Additive genetic variance after admixture

Here, we derive the expected genetic variance after admixture between two source populations (Equation 5 in the main text). Suppose two population (labeled 0 and 1) diverged some time in the past and then admix in proportions f and $1-f$.

Assuming random mating, linkage equilibrium, and no dominance or epistasis (so $V_G = V_A$),

$$V_G = \sum_l 2p_l(1-p_l)a_l^2 = \sum_l \pi_l a_l^2,$$

where π denotes expected pairwise diversity. At a given locus (dropping the l), after admixture the allele frequency is

$$p = fp_0 + (1-f)p_1,$$

so that

$$\begin{aligned}
2p(1-p) &= 2(fp_0 + (1-f)p_1)(1 - fp_0 - (1-f)p_1) \\
&= f^2 2p_0(1-p_0) + (1-f)^2 p_1(1-p_1) + 2f(1-f)(p_0(1-p_1) + p_1(1-p_0)) \\
&= f^2\pi_{0,0} + (1-f)^2\pi_{1,1} + 2f(1-f)\pi_{0,1}.
\end{aligned}$$

Here, $\pi_{i,j}$ is the expected pairwise diversity between two samples, one drawn from population i and the other from population j. If $i=j$, this is pairwise diversity within a single population.

Plugging into the definition for V_G, we get after admixture

$$V_G = f^2 V_{G,0} + (1-f)^2 V_{G,1} + 2f(1-f)\sum_l \pi_{0,1,l}a_l^2.$$

We can write $\pi_{0,1}$ at a given locus in terms of $\pi_{0,0}$, $\pi_{1,1}$, and $F_2(0,1) = (p_0 - p_1)^2$, a measure of single-locus allele frequency differentiation [6]. Then

$$\pi_{0,1} = F_2(0,1) + \frac{1}{2}\pi_{0,0} + \frac{1}{2}\pi_{1,1},$$

and

$$
\begin{aligned}
V_G &= f^2 V_{G,0} + (1-f)^2 V_{G,1} + 2f(1-f)\sum_l \left[F_{2,l}(0,1) + \frac{1}{2}\pi_{0,0} + \frac{1}{2}\pi_{1,1}\right] a_l^2 \\
&= f^2 V_{G,0} + (1-f)^2 V_{G,1} + 2f(1-f)\left[\frac{1}{2}V_{G,0} + \frac{1}{2}V_{G,1} + \sum_l F_{2,l}(0,1) a_l^2\right] \\
&= f V_{G,0} + (1-f) V_{G,1} + 2f(1-f)\sum_i F_{2,l}(0,1) a_l^2.
\end{aligned}
$$

□

D Supplemental Figures

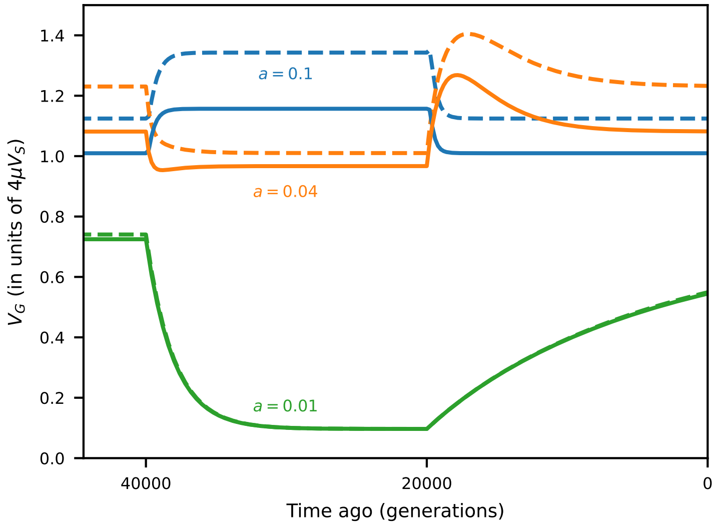

Figure A: Single-population dynamics when V_G is not small. Solid lines: expected V_G assuming $s = a^2/2V_S$. Dashed lines: expected V_G assuming $s = a^2/2(V_S + V_G)$. When mutation rates are large (here, $\mu = 0.01$, $V_S = 1$), V_G is non-negligible compared to V_S. By having to account for V_G in the translation of effect size to selection coefficient, it makes s non-constant if V_G changes over time (due to non-constant demography, for example). In this case, s must be updated regularly, given the current state of the population. Here, the demographic history is a bottleneck followed by a recovery, as depicted in Fig 2A in the main text.

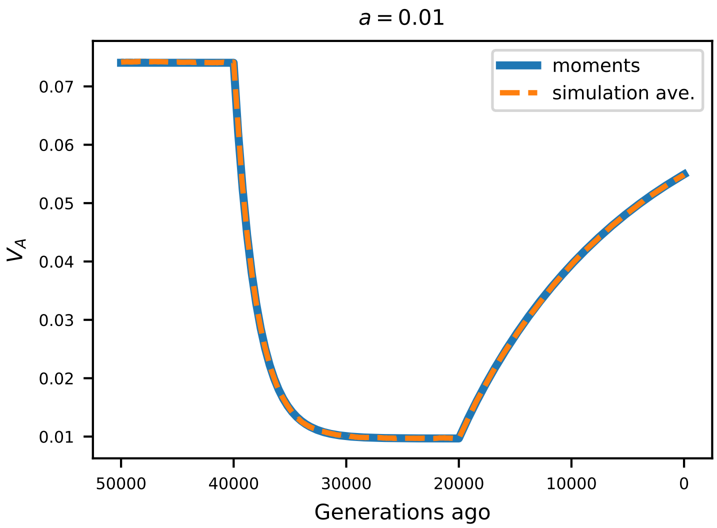

Figure B: Simulations with no linkage and weak effects. Total mutation rate was $\mu = 0.025$ and all mutations had effect sizes $a = \pm 0.01$. The demographic history (bottleneck and recovery) is depicted in Fig 2A in the main text. The predicted trajectory of additive genetic variance using **moments** was found assuming symmetric underdominant selection on trait-affecting alleles, with selection coefficient $s = a^2/2(V_S + V_G)$.

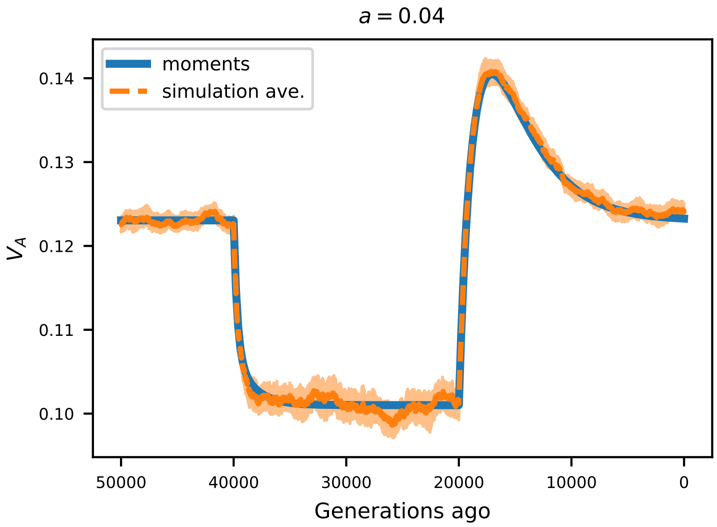

Figure C: Simulations with no linkage and moderate effects. All parameters were consistent with Fig B, but with effect sizes $a = \pm 0.04$.

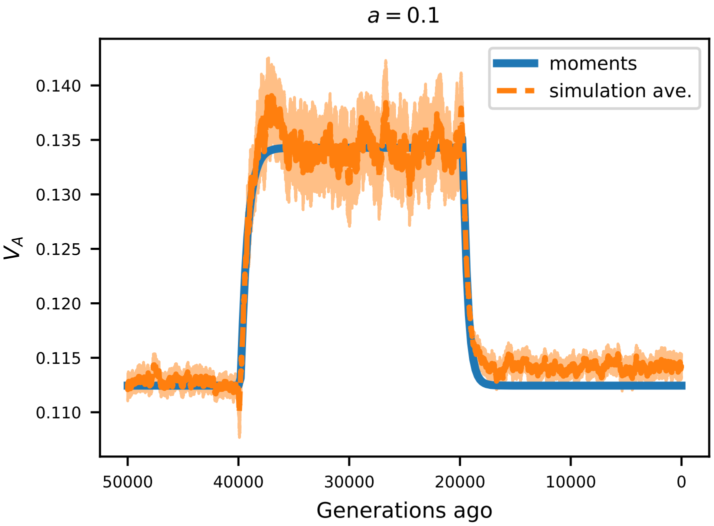

Figure D: Simulations with no linkage and strong effects. All parameters were consistent with Fig B, but with effect sizes $a = \pm 0.1$. In each comparison (weak, moderate, and strong effect sizes), predictions from `moments` closely match observed average additive genetic variance from simulations.

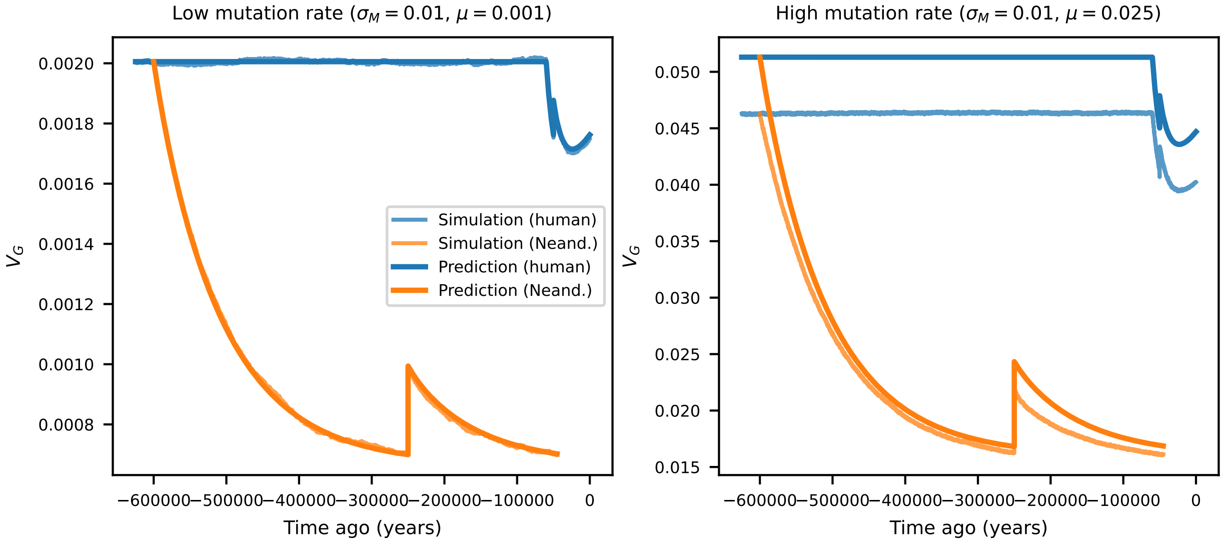

Figure E: With low mutational variance ($V_M = 0.0001$), individual-based simulations using `fwdpy11` closely match our predictions using `moments`, when the mutation rate is small (Left). When the mutation rate is increased, polygenicity increases and unlinked expectations from `moments` deviate from observed V_G in individual-based simulations. In this case, with relatively small mutational variance, the observed V_G is reduced in comparison, consistent with the Bulmber (1971) effect.

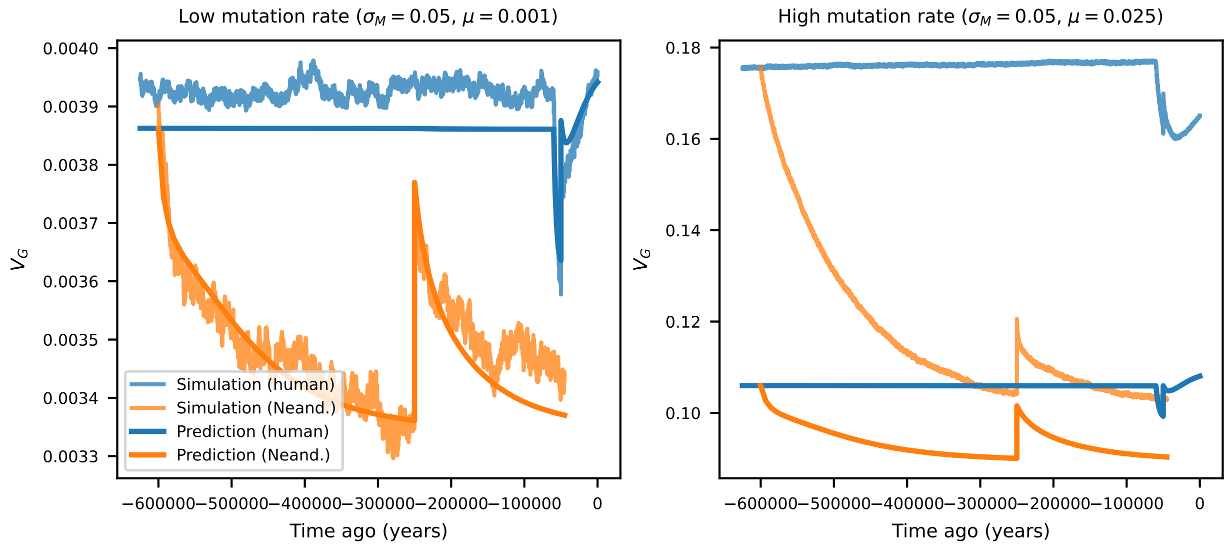

Figure F: With high mutational variance ($V_M = 0.0025$), strong deviations are observed between individual-based simulations and expectations without linkage. When the mutational input is large, observed V_G can be much larger than expectations without linkage, possibly consistent with widespread interference between alleles.

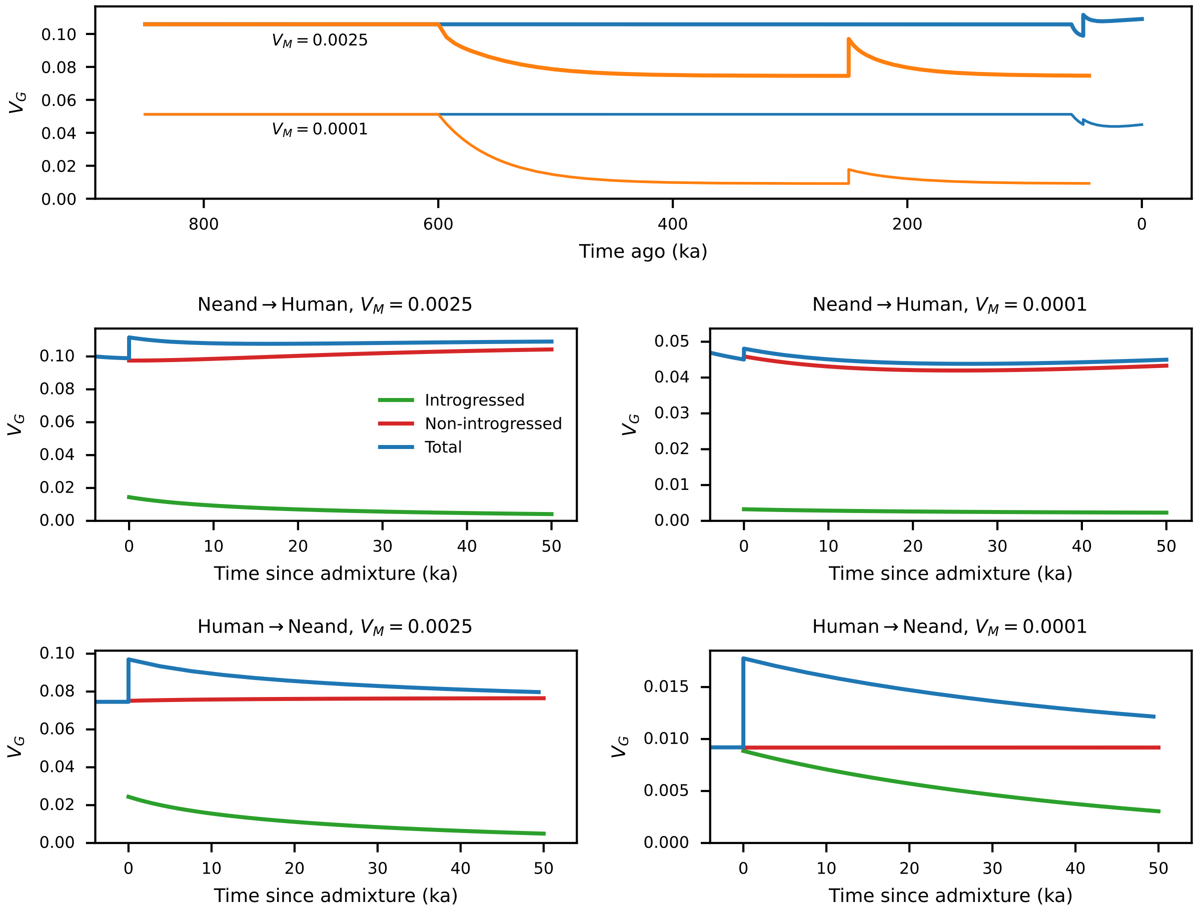

Figure G: Genetic variance of a trait under stabilizing selection in a model with human (blue) and Neanderthal (orange) demography. We consider two mutational variances ($\sigma_M = 0.05$ and 0.01). The demographic model (Fig 4A in the main text) includes 5% admixture from humans to Neanderthals 250 ka, and 2% admixture from Neanderthals to humans 50 ka.

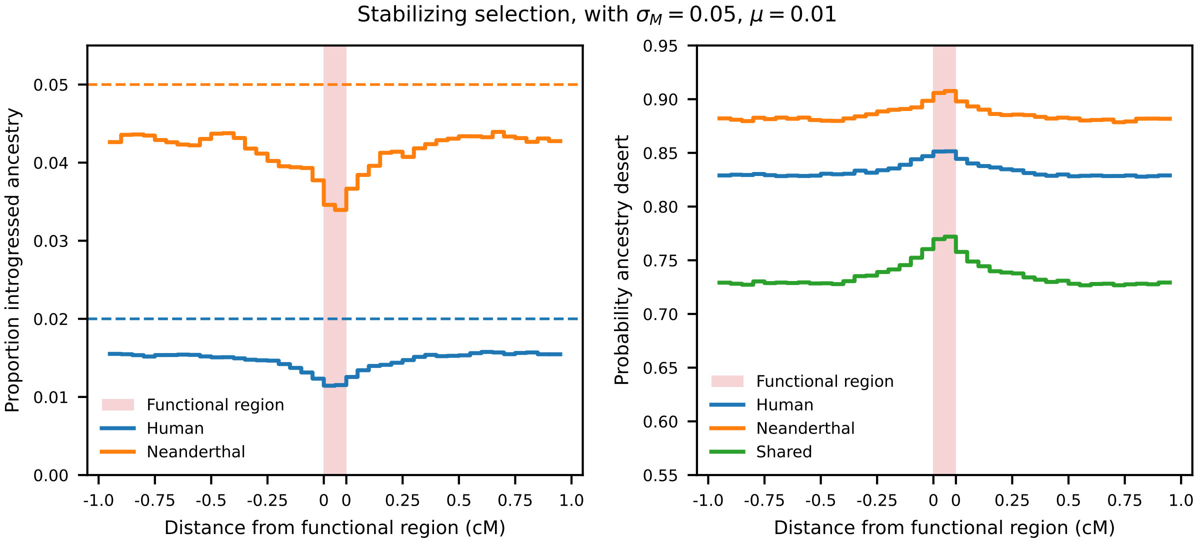

Figure H: Introgressed ancestry and probability of introgression deserts in and around functional regions, from simulations with high mutational variance. The demographic model is shown in Fig 4A in the main text.

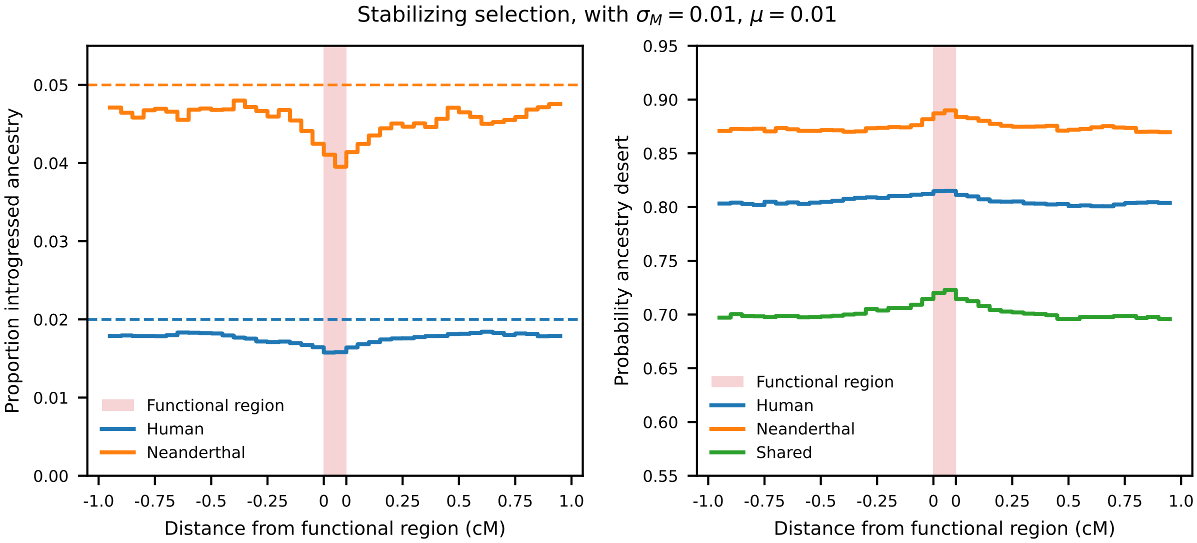

Figure I: Introgressed ancestry and probability of introgression deserts in and around functional regions, from simulations with moderate mutational variance. The demographic model is shown in Fig 4A in the main text.

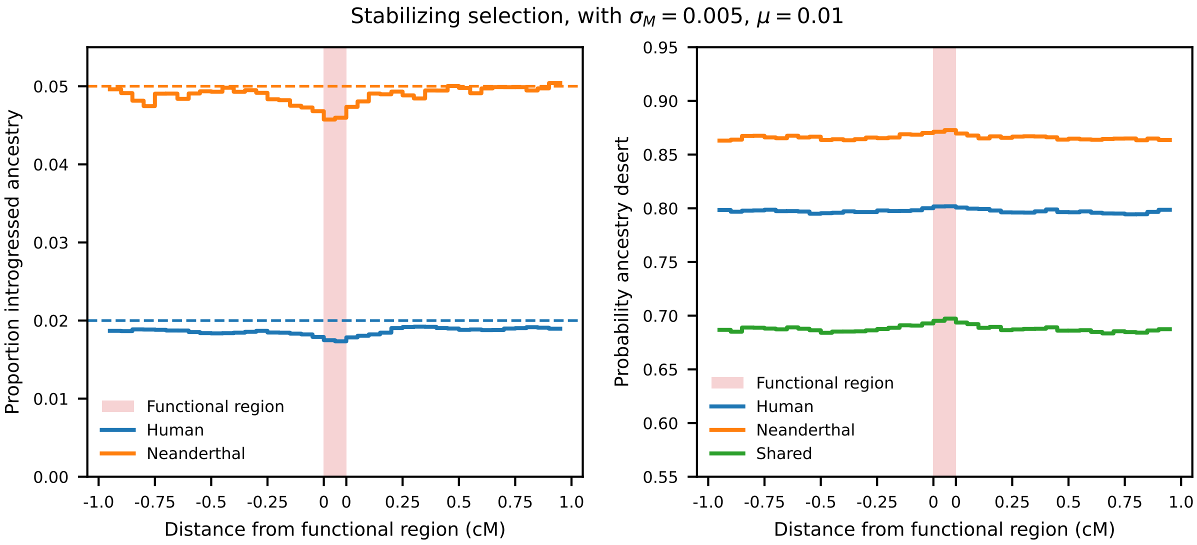

Figure J: Introgressed ancestry and probability of introgression deserts in and around functional regions, from simulations with low mutational variance. The demographic model is shown in Fig 4A in the main text.

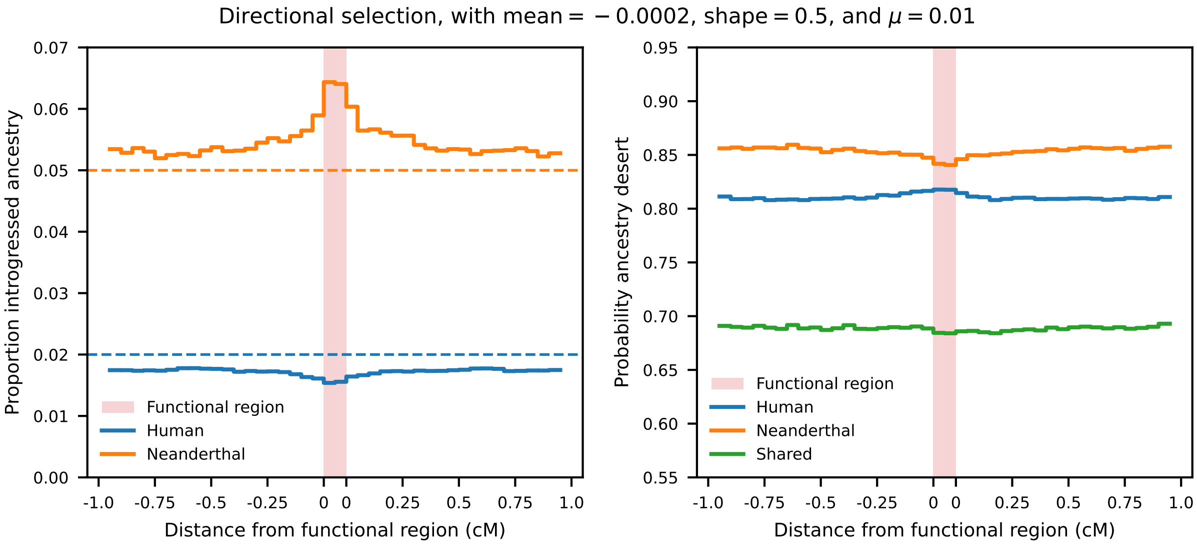

Figure K: Introgressed ancestry and probability of introgression deserts in and around functional regions, from simulations with directional selection. Deleterious mutations were drawn from a gamma distribution with mean -0.0002 and shape 0.05. The demographic model is shown in Fig 4A in the main text.

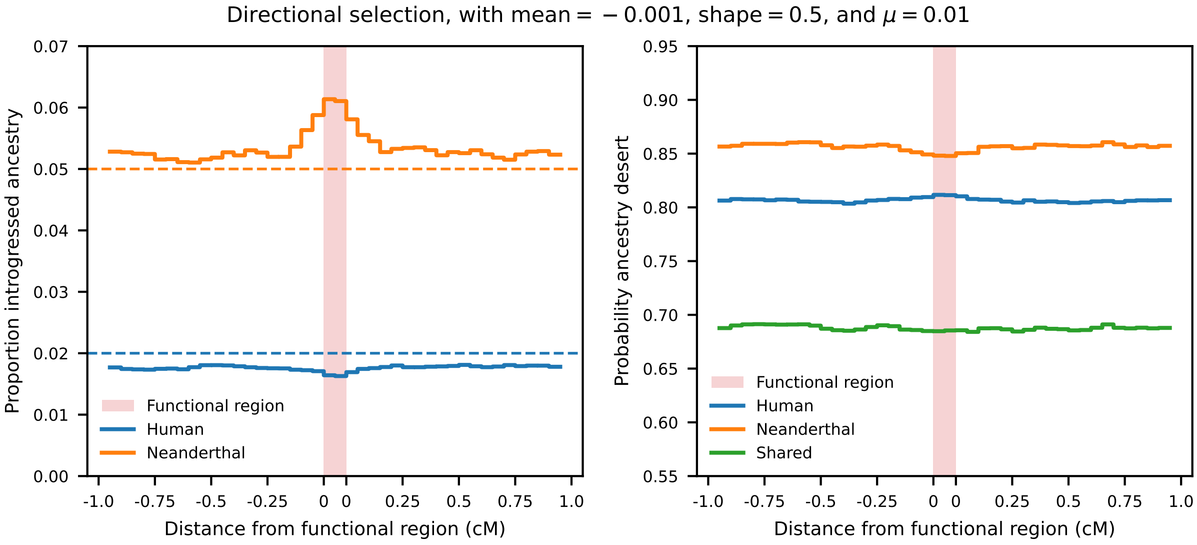

Figure L: Introgressed ancestry and probability of introgression deserts in and around functional regions, from simulations with directional selection. Deleterious mutations were drawn from a gamma distribution with mean -0.001 and shape 0.05. The demographic model is shown in Fig 4A in the main text.

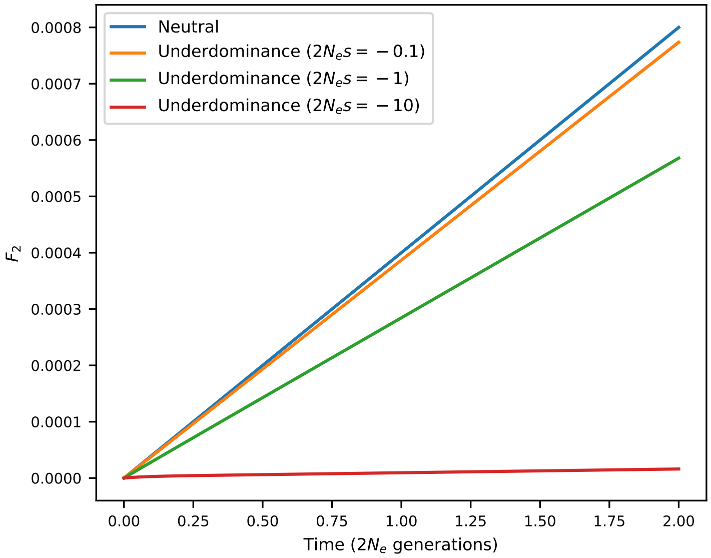

Figure M: Underdominance, like negative selection, reduces expected F_2 compared to neutral divergence.

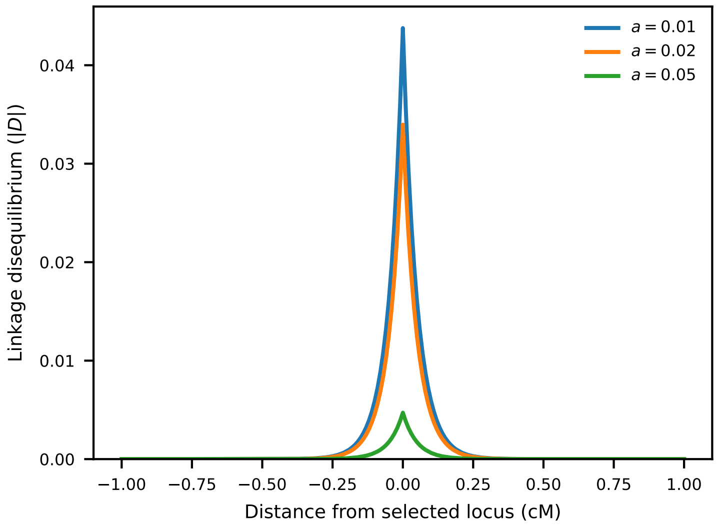

Figure N: Linkage disequilibrium between the a trait-affecting and neutral allele, 2000 generations after admixture. Inititally, the introgression proportion was 0.05. Mutations with stronger effect sizes, and thus stronger selection against them, decrease in frequency more rapidly, leading to reduced LD as measured by $D = Cov(p, q)$ (Fig 5 in the main text).

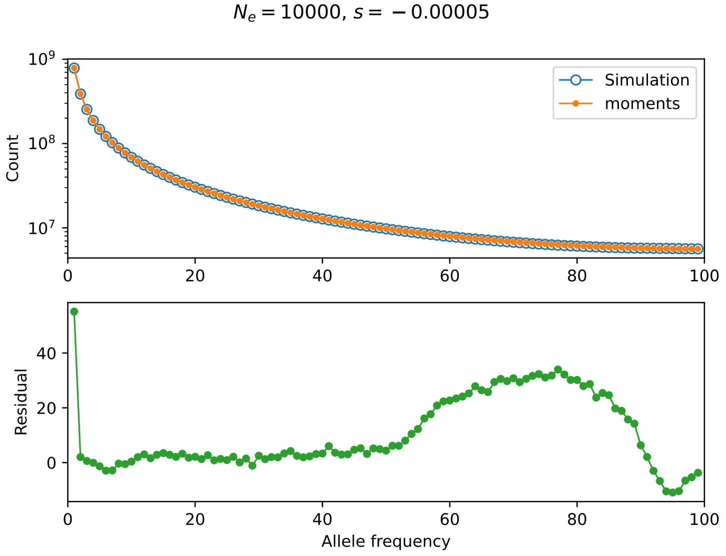

Figure O: Comparison of predicted (`moments`) and simulated SFS with underdominant selection. In this comparison, $N_e = 10^4$ and $s = -5 \times 10^{-5}$, so that the population-size scale selection coefficient $\gamma = -1$. Simulations were performed under a Wright-Fisher model without linkage.

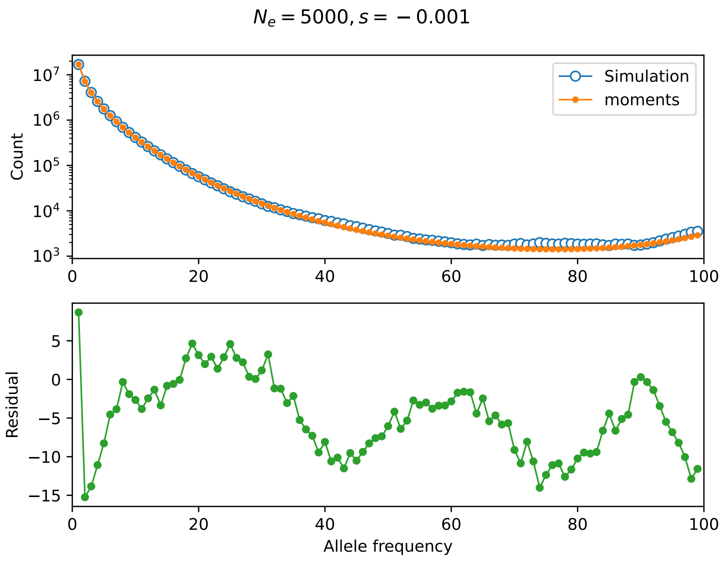

Figure P: Comparison of predicted (`moments`) and simulated SFS with underdominant selection. In this comparison, $N_e = 5000$ and $s = -0.001$, so that the population-size scale selection coefficient $\gamma = -10$. Simulations were performed under a Wright-Fisher model without linkage.

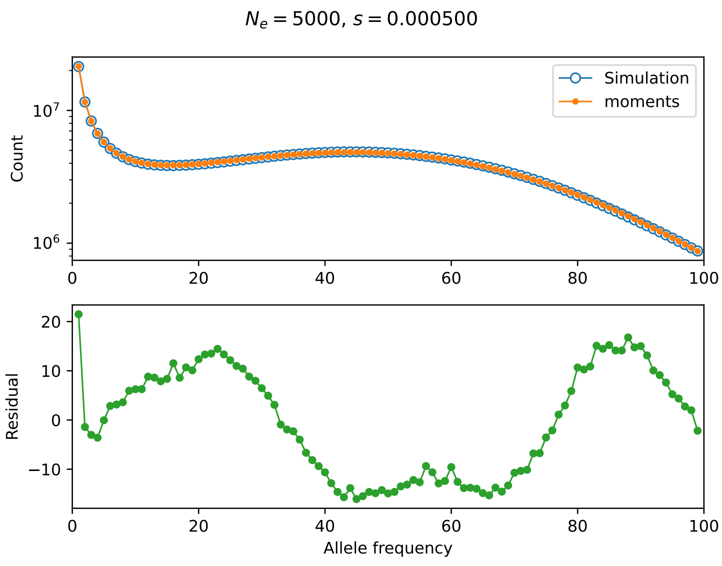

Figure Q: Comparison of predicted (`moments`) and simulated SFS with *over*dominant selection. In this comparison, $N_e = 5000$ and $s = 0.0005$, so that the population-size scale selection coefficient $\gamma = 5$. Thus, with positive selection, heterozygotes are favored. Simulations were performed under a Wright-Fisher model without linkage.

References

[1] Yuval B Simons, Kevin Bullaughey, Richard R Hudson, and Guy Sella. A population genetic interpretation of GWAS findings for human quantitative traits. *PLoS biology*, 16(3):e2002985, 2018.

[2] Sherif Negm and Carl Veller. The effect of long-range linkage disequilibrium on allele-frequency dynamics under stabilizing selection. *bioRxiv*, pages 2024–06, 2024.

[3] Julien Jouganous, Will Long, Aaron P Ragsdale, and Simon Gravel. Inferring the joint demographic history of multiple populations: beyond the diffusion approximation. *Genetics*, 206(3):1549–1567, 2017.

[4] Laura Katharine Hayward and Guy Sella. Polygenic adaptation after a sudden change in environment. *Elife*, 11:e66697, 2022.

[5] Ivan Krukov and Simon Gravel. Taming strong selection with large sample sizes. *bioRxiv*, page 2021.03.30.437711, 2021.

[6] Benjamin M Peter. Admixture, population structure, and F-statistics. *Genetics*, 202(4):1485–1501, 2016.
